# Shiga Toxins as Multi-Functional Proteins: Induction of Host Cellular Stress Responses, Role in Pathogenesis and Therapeutic Applications

**DOI:** 10.3390/toxins8030077

**Published:** 2016-03-17

**Authors:** Moo-Seung Lee, Sunwoo Koo, Dae Gwin Jeong, Vernon L. Tesh

**Affiliations:** 1 Infection and Immunity Research Center, Korea Research Institute of Bioscience and Biotechnology, 125 Gwahak-ro, Daejeon 34141, Korea; 2 Research Center for Viral Infectious Diseases and Control, Korea Research Institute of Bioscience and Biotechnology, 125 Gwahak-ro, Daejeon 34141, Korea; dgjeong@kribb.re.kr; 3 Department of Neuroscience and Experimental Therapeutics, Texas A & M University Health Science Center, Bryan, TX 77807, USA; 4 Department of Microbial Pathogenesis and Immunology, Texas A & M University Health Science Center, Bryan, TX 77807, USA; tesh@medicine.tamhsc.edu

**Keywords:** Shiga toxins, Shiga toxin type 1 and 2, Shiga toxin-producing *Escherichia coli*, hemolytic uremic syndrome, signaling pathways, cancer therapeutics

## Abstract

Shiga toxins (Stxs) produced by Shiga toxin-producing bacteria *Shigella dysenteriae* serotype 1 and select serotypes of *Escherichia coli* are primary virulence factors in the pathogenesis of hemorrhagic colitis progressing to potentially fatal systemic complications, such as hemolytic uremic syndrome and central nervous system abnormalities. Current therapeutic options to treat patients infected with toxin-producing bacteria are limited. The structures of Stxs, toxin-receptor binding, intracellular transport and the mode of action of the toxins have been well defined. However, in the last decade, numerous studies have demonstrated that in addition to being potent protein synthesis inhibitors, Stxs are also multifunctional proteins capable of activating multiple cell stress signaling pathways, which may result in apoptosis, autophagy or activation of the innate immune response. Here, we briefly present the current understanding of Stx-activated signaling pathways and provide a concise review of therapeutic applications to target tumors by engineering the toxins.

## 1. Introduction

Shiga toxin (Stx) is a protein exotoxin expressed by the Gram-negative bacteria *Shigella dysenteriae* serotype 1. The toxin is named after Dr. Kiyoshi Shiga, a Japanese bacteriologist who first identified bacteria that came to be called “Shiga’s bacillus” as the causative agent of a widespread outbreak of “red diarrhea” (dysentery) in 1897 [1]. The work of many microbiologists in the early 20th century defined cytotoxic, neurotoxic and enterotoxic activities in extracts prepared from the organism variously known as Shiga’s bacillus, *Bacillus dysenteriae*, *Shigella shigae* and now classified as *S. dysenteriae* serotype 1. In 1980, the publication of purification protocols for Shiga toxin from *Shigella dysenteriae* serotype 1 greatly facilitated the study of the toxin [2,3]. In 1977, culture filtrates prepared from some *E. coli* strains causing diarrhea in humans were shown to produce a cytotoxin capable of killing Vero cells [4]. Based on this observation, the cytotoxin was referred to as Vero cytotoxin or Verotoxin. Shortly thereafter, it was reported that a Shiga-like toxin was produced by *E. coli* O157:H7 strain 933 that had caused an outbreak of hemorrhagic colitis in the United States [5]. The toxin was capable of killing Vero and HeLa cells, was lethal when administered to mice and caused fluid accumulation in ligated rabbit ileal loops. Subsequently, *E. coli* O157:H7 strain 933 was shown to be lysogenized by two toxin-converting bacteriophages encoding toxins that were antigenically similar to Shiga toxin [5]. Recently, Mora *et al.* [6] reported that over 470 *E. coli* serotypes causing disease in humans harbor bacteriophages encoding genetic variants of Shiga toxin expressed by *S. dysenteriae* serotype 1. Collectively, these *E. coli* are called Shiga toxin-producing *E. coli* (STEC) or Verotoxin-producing *E. coli* (VTEC), and the terms Shiga toxins or Verotoxins are used to describe the same toxins [7].

*Shigella dysenteriae* serotype 1 and STEC are major public health concerns in developed and developing countries due to the severity of the diseases they cause. Infections with *S. dysenteriae* serotype 1 (shigellosis) and STEC may result in bloody diarrhea (hemorrhagic colitis) and the subsequent development of life-threatening sequelae, including acute renal failure and neurological abnormalities, such as seizures, paralysis, blindness and death [8]. The young and elderly are most vulnerable to developing life-threatening complications following infection with *S. dysenteriae* serotype 1 or STEC [9]. The acute renal failure that may follow hemorrhagic colitis is the main feature of hemolytic uremic syndrome (HUS). HUS is defined by a triad of symptoms: thrombocytopenia, microangiopathic hemolytic anemia and acute renal failure. Readers are referred to several excellent reviews on the extra-intestinal complications that may follow the ingestion of Shiga toxin-producing bacteria [10,11,12]. Human-to-human transmission via the fecal-oral route is the primary mode of transmission of *S. dysenteriae* serotype 1. In the environment, ruminant animals serve as reservoirs for STEC, with the primary mode of transmission to humans mainly involving fecal contamination of drinking water or under-chlorinated swimming pool water and foods, such as under-cooked meat products, unwashed vegetables and unpasteurized dairy products. STEC may also be transmitted through petting animals [13,14]. In developed countries, STEC constitute a heightened public health concern because of the potential for contaminated foods to be distributed on a nationwide basis. Multi-state outbreaks in the U.S. involving contaminated beef products or vegetables highlight this potential [15]. In 1999, the Centers for Disease Control and Prevention (CDC) estimated that approximately 73,000 cases of hemorrhagic colitis occurred annually in the United States due to *E. coli* O157:H7, with 37,000 cases caused by non-O157 serotype STEC infections [16]. Of these cases, approximately 2000 required hospitalization, with 60–100 mortalities [16]. In 2013, the annual incidence in the U.S. of *E. coli* O157:H7-associated illnesses was estimated to be 63,153 cases, while illnesses caused by STEC serotypes other than O157 were estimated to be 112,752 cases [17]. These epidemiological studies highlight the rapid emergence of *E. coli* non-O157:H7 serotypes as mediators of disease. In 2011, a widespread outbreak of gastroenteritis occurred in Europe associated with the ingestion of STEC-contaminated fenugreek or lentil sprouts [18]. This outbreak was particularly problematic for two reasons: (i) the causative agent was *E. coli* O104:H4, a serotype previously characterized as an enteroaggregative *E. coli*; and (ii) a high percentage of patients with diarrhea subsequently developed HUS (845/3816 cases) [18,19]. The annual economic costs of *E. coli* O157 infections in the United States alone, including costs of medical care, lost productivity and premature death, has been estimated at $405 million (in 2003 dollars) [20]. Shigellosis is also a major public health problem, especially in developing countries, with an estimated incidence of 1.1 million fatal cases per year and annual cases worldwide estimated at 160 million [21,22]. The infectious dose of *S. dysenteriae* serotype 1 or STEC sufficient to cause disease in humans may be as low as 10–100 microbes [23,24]. Currently, there are no satisfactory vaccines to prevent diseases caused by Shiga toxin-producing bacteria and no effective therapeutic regimens to interrupt disease progression once the toxins interact with susceptible human cells.

Shiga toxins (Stxs) are key virulence factors expressed by *S. dysenteriae* serotype 1 and STEC; that is, toxin production has been shown to exacerbate intestinal damage and cause extra-intestinal complications involving the kidneys and CNS. Many of the systemic complications seen in humans can be reproduced by the administration of purified toxins into animals. There are two main categories of Stxs produced by STEC, Shiga toxin type 1 (Stx1) and Shiga toxin type 2 (Stx2), based on their antigenic similarity to the prototypical Shiga toxin expressed by *S. dysenteriae* serotype 1 [25,26]. All Stxs contain a pentameric ring of identical B-subunits non-covalently associated with a single A-subunit (see Section 2 below). The toxins cross the intestinal epithelium through processes that may involve bacterial invasion and epithelial cell destruction (*S. dysenteriae* serotype 1) or transcytosis of toxins across intestinal epithelial cells [27,28]. The toxins circulate in the bloodstream, perhaps bound through low-affinity interactions with the surface of carrier cells, such as neutrophils or blood monocytes [29,30]. Cellular microvesicles (MVs) [31] derived from platelets, erythrocytes and leukocytes are detected in plasma collected from pediatric HUS patients infected with *E. coli* O157:H7 and adult HUS patients infected with *E. coli* O104:H4 [32,33]. A significant fraction of circulating monocytes and neutrophils in HUS patients were shown to be associated with CD41^+^ platelet-derived MVs [33]. Recently, Ståhl *et al.* [34] detected MVs derived from platelets and leukocytes containing Stx2 in plasma of patients during the acute phase of HUS. Furthermore, Stx2-containing MVs were also detected adjacent to or within glomerular endothelial cells in a renal cortical biopsy from an HUS patient [34]. Taken together, these findings suggest that once in the microvasculature serving target organs (primarily the kidneys and brain), the toxins may transfer from leukocytes or MVs associated with leukocytes to susceptible endothelial cells via high-affinity interactions with a membrane glycolipid receptor, globotriaosylceramide (Gb_3_, also known as CD77 or P^k^ blood group antigen). Once bound to the receptor (Gb_3_), the toxins are internalized and undergo retrograde intracellular trafficking. The receptor-mediated entry of Stxs into host cells has been extensively studied. Gb_3_ cross-linking through interaction with the pentameric B-subunits is thought to trigger receptor-mediated endocytosis [35,36,37]. Following internalization, the toxins are sequentially delivered from an early endosome to the trans-Golgi network, through the Golgi apparatus, to the endoplasmic reticulum (ER). This process is known as retrograde transport, as the toxins appear to utilize, in a Gb3-dependent retrograde manner, the host cell machinery involved in the export of secreted or membrane-localized proteins [38]. During transport to the ER, Stx A-subunits dissociate from the B-subunits through a processing mechanism involving proteolysis and disulfide bond reduction [39,40,41]. Fragments of the A-subunits then associate with host ER intraluminal chaperones ERdj3/HEDJ, GRP94 and BiP, followed by retrotranslocation across the ER membrane into the cytosol [42,43,44,45]. Recent studies suggest that Stxs utilize the host cell endoplasmic reticulum-associated protein degradation (ERAD) machinery to facilitate translocation into the cytoplasm [46,47,48]. The ERAD process normally targets misfolded host proteins in the ER to the proteasome for degradation. Once in the cytoplasm, however, toxin A-fragments appear to re-fold into their active conformation [49]. This observation indicates that, as is the case for retrograde transport, the toxins may utilize normal host cell machinery for intoxication. Delivery of the toxins to the ER and retrotranslocation of the processed A-subunits into the cytoplasm result in host cell protein synthesis inhibition, activation of the ribotoxic stress and ER stress responses and, in some cases, the induction of apoptosis, autophagy and increased expression of pro-inflammatory cytokines and chemokines [50]. Remarkably, many types of cancer cells overexpress Gb_3_ on their surface, and therefore, the binding of toxins or the non-toxic pentameric Stx B-subunits coupled to anti-cancer agents has been explored for targeted cancer therapeutics [51,52,53,54] This paper will provide a concise review of the multi-functionality of Stxs.

## 2. Stx Structure and Receptor Interaction

**Figure 1:**
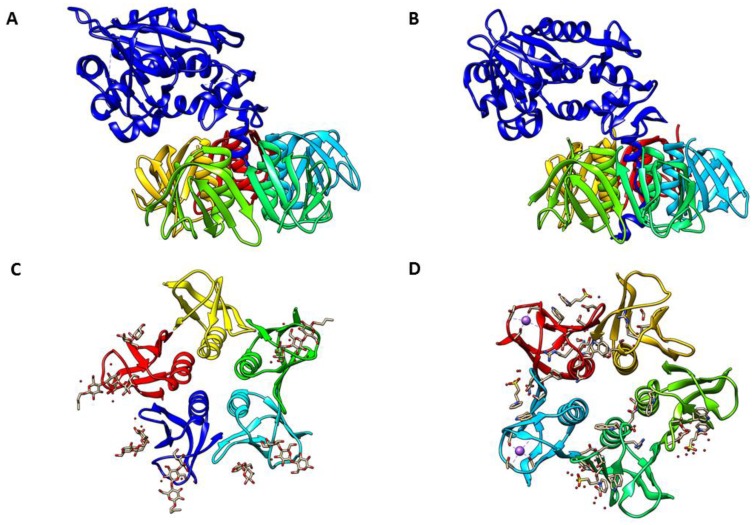
Crystal structure of Shiga toxin. (**A**) Shiga toxin type 1 (PDB #1DM0); (**B**) Shiga toxin type 2 (PDB #1R4P); (**C**) Shiga toxin 1 B-subunit with Gb3 receptor (PDB #1BOS); (**D**) Shiga toxin 2 B-subunit with Gb3 receptor (PDB #1R4P, deletion of A-subunit). In (**A**,**B**), A-subunits are shown in dark blue, with B-subunits shown in different colors; in (**C**,**D**), individual B-subunits are shown in different colors. PDB files of all structures were obtained from RCSB PDB (Research Collaboratory for Structural Bioinformatics Protein Data Base, www.rcsb.org) and compiled PDB files with Chimera 1.10.2 (UCSF Chimera, www.cgl.ucsf.edu/chimera).

The X-ray crystal structures of Stx holotoxins, Stx1 and Stx2, at 2.5-Å and 1.8-Å resolution, respectively, have been solved and their active sites defined from electronic densities of the crystallization solutions (Figure 1A,B) [55,56]. All members of the Shiga toxin family share the same structural configuration comprised of an enzymatically-active A-subunit of approximately 32 kDa non-covalently linked with five identical B-subunits, each B-subunit protein being ~7.7 kDa in size [49]. Thus, Shiga toxins are part of a larger class of structurally-conserved bacterial protein toxins, termed AB_5_ toxins, that includes *Vibrio cholerae* cholera toxin and the *E. coli* heat-labile enterotoxins [57,58,59]. In the crystallographic analyses of the holotoxins, the homopentameric B-subunits form a ring with the carboxy terminus of the A-subunit inserted within the central pore [55,56]. A major structural difference in Stx1 and Stx2 is that in contrast to the Stx1 A-subunit, the C-terminus of the Stx2 A-subunit forms an α-helix after passing through the central pore of the B-subunits. The A-subunits of Stxs possess a highly specific *N*-glycosidase activity to remove a single adenine base, located at position 4324 in the rat, from the ribose-phosphate backbone of the 28S rRNA component of 60S eukaryotic ribosomes [60,61]. Stx A-subunits are cleaved asymmetrically by furin or a furin-like protease into two peptides, A_1_ (~27.5 kDa) and A_2_ (~4 kDa), that are held together by a disulfide bond [39]. The enzymatically-active A_1_-fragment is released by reduction of the disulfide bond upon exposure to the reducing conditions in the ER lumen of target cells. Mutagenesis studies showed that the A_1_-fragment active site of *N*-glycosidase function includes position 167 (glutamic acid) [62], which was necessary to remove an adenine base in domain VI of 28S ribosomal RNA of eukaryotic ribosomes [60,63]. It has been reported that the B-subunits of these Stxs bind to the Gb_3_ receptors, while these weakly bind to the neutral glycolipid globotetraosylceramide (Gb_4_) [64,65]. Recently, however, Gallegos *et al.* reported that both Stx1 and Stx2 bound to Gb_3_ and Gb_4_ *in vitro* with comparable affinities [66]. In human neutrophils, toll-like receptor (TLR) 4 has recently been revealed as a receptor recognizing the A-subunits of Stxs [67]. Based on the crystal structure of B-subunits, each Stx1 and Stx2 B-subunit monomer contains three distinct binding sites, numbered sites 1–3, for the glycan component of Gb_3_ (Figure 1C,D) [68,69,70]. Thus, each homopentameric ring of B-subunits may contain a total of 15 Gb_3_ binding sites [71]. Further , site 2 in each B-subunit demonstrated higher occupancy than site 1, and there was less interaction detected with site 3 [72]. Based on the mutational studies that confirmed the primary role of site 2 in Stx1 binding [73], the interaction of site 2 with Gb_3_ on the target cell surface plays an essential role for the toxicity of Stx1 and Stx2 [74].

The binding affinity of homopentameric B-subunits (15 binding sites, binding constant = 10^9^ M^−1^) is higher than that of monomeric B subunits (three binding sites, binding constant = 10^3^ M^−1^), suggesting that multiple binding sites dramatically enhance the binding affinity [75]. Most of the structural and binding data have been collected by using Stx1. Recently, Jacobson *et al.* [69] reported the first crystal structure of pentameric B-subunits of Stx2 bound to a disaccharide analog of Gb_3_ and demonstrated that sites 1 and 2 are functional, and the primary binding site was site 2, as it was in experiments using Stx1. Interestingly, the binding affinity of carbohydrates for site 2 in Stx2 was higher than that interaction in Stx1 [76]. Conrady *et al.* reported that pentameric B-subunits of Stx1 were more stable than that of Stx2 before the crystal structure of Gb_3_ bound Stx2 was solved [77]. Site 3 in Stx2 B-subunits is partially blocked by the C-terminus of the A_2_-fragment, leading to inactive binding sites until the A-subunit is processed within the ER [55]. Taken together, these data may indicate that there are differences in the receptor binding mechanisms of Stx1 and Stx2, and differences in receptor binding preferences between Stx1 and Stx2 may contribute to different toxicities observed between the toxins [78].

Stx holotoxin molecules are internalized into the cells through clathrin-dependent or clathrin-independent endocytosis pathways depending on the cell type examined [79]. It has been reported that the clathrin-dependent process is the most common pathway of cellular uptake following receptor binding by the toxins [79]. However, the full spectrum of Stx cellular uptake mechanisms remains to be characterized. For example, Malyukova *et al.* [80] demonstrated that macropinocytosis is an alternative pathway for the uptake of Stxs into cells that do not express Gb_3_. The unique mechanisms of internalization of B-subunits of Shiga toxin (StxB) through Gb_3_ receptors, binding the pentameric form of StxB with up to 15 Gb_3_ receptors with high affinity [75], low immunogenicity *in vivo* [81] and minimization of lysosomal degradation through the retrograde trafficking, strongly encouraged many researchers to exploit this protein as a targeted cancer therapeutic agent, such as drug delivery and high intensity tumor cell imaging. Indeed, StxB-based anticancer agents are currently being developed as a targeted delivery cargo of therapeutic molecules [54,82]. Most recently, overexpression of Gb_3_ was detected in 57.9% of breast cancer patients (62 of 107 patients). Interestingly, Gb_3_ expression is enhanced in lymph node metastases (40% of primary tumors) and positively correlated with estrogen receptor expression, which is the most recent clinical evidence of the expression pattern of Gb_3_ in cancer patients [83].

## 3. Stx Induces Multiple Signaling Pathways

**Figure 2:**
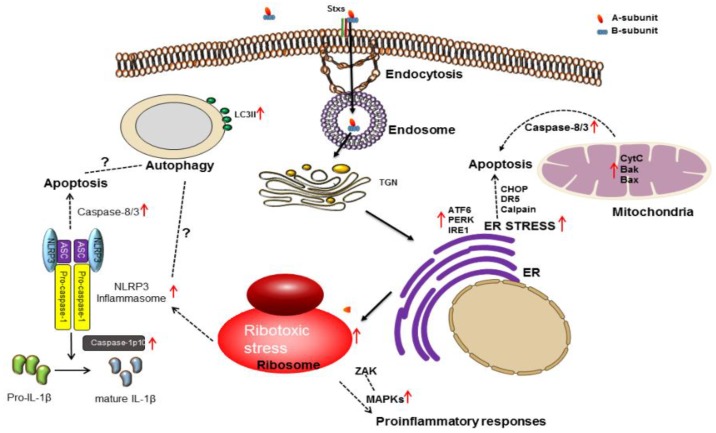
Host cell responses (ribotoxic stress, ER stress, inflammation, autophagy and apoptosis) induced by Stxs, following the membrane invagination-mediated endocytosis via toxin receptor Gb3 on the cell surface.

In addition to mediating protein synthesis inhibition, Stxs are multi-functional bacterial proteins activating a number of signaling pathways, including those associated with the activation of the ribotoxic stress response and endoplasmic reticulum (ER) stress. Signaling through these pathways may lead to cell death via apoptosis or autophagy or activation of innate immunity associated with proinflammatory cytokine/chemokine production (Figure 2). Cytokine and chemokine production may contribute to tissue damage in the colon and the development of HUS and CNS complications. Signaling pathways activated by Stxs, linked to the eventual induction of apoptosis in epithelial, endothelial, lymphoid and myeloid cells *in vitro*, may contribute to tissue damage in multiple organs.

### 3.1. Ribotoxic Stress Response

As previously discussed, the binding of Stxs to Gb_3_ triggers toxin internalization, followed by retrograde transport to the trans-Golgi network and ER, where processed A-subunit fragments ultimately reach the cytosol (Figure 2). Subsequently, the *N*-glycosidase enzymatic activity of Stx A_1_-fragments cleaves a single adenine residue from 28S rRNA of the 60S ribosomal subunit. The depurination reaction involves an unpaired adenine residue located in a region of non-Watson-Crick base-pairing called the α-sarcin/ricin loop (so-called because the ribosome-inactivating toxins α-sarcin and ricin also act on the same adenine residue). This depurination process leads to loss of elongation factor binding and inhibition of protein synthesis [57,64,84]. Following depurination, the alteration of ribosomes by Stxs may result in the initiation of proinflammatory and proapoptotic signaling cascades via activation of mitogen-activated protein kinases (MAPKs). This process was termed the ribotoxic stress response after Iordanov *et al.* [85] showed that certain protein synthesis inhibitors, including ricin, anisomycin and α–sarcin, mediating site-specific modifications to the ribosomal peptidyl transferase center, activated c-Jun N-terminal kinase 1 (JNK1). Thus, the work of Iordanov *et al.* [85] suggested that Stxs should activate stress-activated protein kinase cascades, as well. However, other molecules, such as cycloheximide, emetine, T-2 toxin, pactamycin and puromycin, capable of effectively inhibiting protein synthesis, failed to induce the ribotoxic stress response through activation of JNK1. Thus, eukaryotic ribosomes possess a selective stress sensory function leading to the activation of cell stress responses. It was subsequently shown using human epithelial and monocytic cell lines that Stx-mediated modification of the ribosome triggered not only the activation of the c-Jun N-terminal kinases (JNKs), but also activated the p38 mitogen-activated protein kinase (p38 MAPK) and extracellular-signaling regulated kinase (ERK) pathways [86,87,88]. These studies supported the necessity of Stx A_1_-fragment retrotranslocation and action on the ribosomal peptidyl transferase center for MAPK activation. The complete signaling cascades linking alterations in ribosomal function with MAPK activation remain to be fully characterized (Figure 2). However, the upstream kinase, double-stranded RNA-activated protein kinase R (PKR; a serine/threonine kinase) activated by damaged ribosomes through interaction with two dsRNA-binding domains has been identified as a mediator of the ribotoxic stress response [89]. Zhou *et al.* showed that PKR participates in signaling during the ribotoxic stress response induced by ribosome-inactivating proteins ricin, Stxs or the fungal trichothecene toxin deoxynivalenol (DON) [90]. Moreover, IL-8 expression induced by DON through the ribotoxic stress response required a second kinase, hematopoietic cell kinase (Hck), which associates with the 40S ribosomal subunit and triggers activation of ASK1, MKK3/6 and p38 MAPK in mononuclear phagocytes [91]. The observation on the requirement to generate a scaffold for the direct binding of JNK and p38MAPK to double-stranded RNA binding protein E3L suggests that PKR is an immediate sensor of ribosomal alterations [92]. Jandhyala *et al.* [93] have shown that the MAP3K zipper sterile-α-motif kinase (ZAK) is involved in the ribotoxic stress response. A ZAK inhibitor and ZAK siRNAs blocked Stx2- and ricin-mediated activation of stress-activated protein kinases and partially protected Hct8 and Vero cells from Stx2-induced apoptosis. In contrast to these intestinal and renal epithelial cell lines, the Ramos Burkitt’s lymphoma cell line appears to express basal levels of activated p38 MAPK, and Stx1 treatment did not increase p38 MAPK activation above basal levels. Inhibitors of p38 MAPKs actually increased apoptosis induced by Stx1 treatment of Burkitt’s lymphoma cells [94]. Taken together, these data suggest that there may be multiple signaling pathways for the activation of the ribotoxic stress response, some of which may be cell-type specific. The complete characterization of upstream regulators involved in the ribotoxic stress response elicited by Stxs may be required to develop effective interventional therapeutic approaches to prevent the progression of disease caused by the toxins.

### 3.2. ER Stress

The ER is a multifunctional organelle, where, following synthesis by ribosomes, nascent polypeptides are folded correctly and the proteins transported to other parts of the cells or secreted. The ER is also critical in regulating intracellular calcium levels. However, overloading the ER lumen with truncated or misfolded proteins leads to ER stress and initiation of the unfolded protein response (UPR) to cope with the stress [95,96,97]. If activation of the UPR fails to clear the cause of ER stress, apoptotic cell death may follow (reviewed by Szegezdi *et al.* [98]). The chaperone binding immunoglobulin protein (BiP, also known as GRP78) is typically associated with the following three ER membrane proteins to sense the levels of unfolded proteins: RNA-dependent protein kinase-like ER kinase (PERK), inositol-requiring ER to nucleus signal kinase-1 (IRE1) and activating transcription factor-6 (ATF6). In host-pathogen interface studies, the ER stress response has been reported to be activated, and the subsequent triggering of the UPR may contribute to a cytoprotective response directed against invading microorganisms, including various viruses or bacteria [99,100,101,102]. Of note, Stx molecules are thought to associate with ER-resident chaperone proteins HEDj/ERdj3, Grp94 and BiP, suggesting that during retrotranslocation, Stx A_1_-fragments may exist in a transient unfolded state [43,44]. Using monocytic THP-1 cells, Lee *et al.* [103] first demonstrated that Stx1 was capable of inducing ER stress and activating the three proximal UPR effectors, PERK, IRE1 and ATF6, involved in the immediate detection of unfolded proteins (Figure 2). In addition to monocytic cells, ER stress signaling activated by Stxs has been investigated in other cell types. Using the immortalized human proximal tubule epithelial cell line HK-2 as an *in vitro* model of Stx-mediated renal damage, Stx1 and Stx2 were shown to differentially activate non-overlapping sensors of ER stress. The intoxicated cells were susceptible to the cytotoxic action of Stxs with significant cleavage of poly(ADP-ribose) polymerase (PARP), an enzyme primarily involved in DNA repair and induction of programmed cell death [104]. Parello *et al.* observed that prolonged renal ER stress was capable of contributing to apoptosis in murine models of Stx2-induced kidney injury, although the downregulation of ER stress induced by Stx2 was not sufficient to prevent renal cytotoxicity in mice [105]. When Caco-2 cells, a cultured line of human enterocytes, were exposed to Stx2, autophagic cell death preceding apoptosis was promoted through ER stress [106]. The precise relationships of ER-to-cytoplasm retrotranslocation processes in inducing ER stress and apoptosis in cells maintained *in vitro vs.* Stx-induced organ injury leading to organ failure *in vivo* remain to be fully characterized. However, Stx trafficking to the ER and transport of processed toxin across the ER membrane are critical steps in toxin-mediated cell death, and additional investigations are needed to define reasonable therapeutic targets in toxin trafficking pathways to intervene in disease progression.

### 3.3. Apoptosis

Apoptosis (programmed cell death) is a form of cell death that ensues following activation of intracellular signaling pathways in response to a variety of cell stressors. The ability of Stxs to induce apoptosis may play an important role in causing intestinal damage, as well as extra intestinal complications, such as HUS and vascular damage in the CNS. For example, in HUS, extensive damage to glomeruli and renal tubular epithelial cells was observed with pyknotic nuclei and sloughing of cells into the tubule lumina [107]. Cellular apoptotic characteristics, including cytoplasmic condensation, nuclear chromatin changes, formation of apoptotic bodies and DNA fragmentation, were observed in Vero cells treated with Stx1 by utilizing light microscopy and DNA-agarose gel electrophoresis with ethidium bromide staining [108]. Moreover, apoptotic nuclei localized to tubular epithelial cells and glomerular cells were observed after TUNEL staining of renal cortical tissues [109]. Using renal biopsies from seven HUS patients, te Loo *et al.* [110] subjected the tissue samples to dual staining with TUNEL and SC-35 (a dye to label for RNA synthesis and splicing factor to avoid non-specific TUNEL positive staining) and showed that 80% of apoptotic cells were detected in tubules and 20% in glomeruli. Smith *et al.* [88] linked the apoptotic programmed cell death pathway with signaling through the ribotoxic stress response triggered by exposure to Stx1 in the human epithelial cell line Hct8. Functionally-active Stx1 holotoxin, but not an enzymatically inactivated Stx1 mutant, triggered caspase-3 (executioner caspase) activation and nuclear fragmentation (karyorrhexis). Hct8 cells were partially protected from apoptosis with reduced activity of caspase-3 and DNA fragmentation when the cells were stimulated with a p38 MAPK inhibitor prior to treatment with Stx1 [88]. In Hct8 and Vero cells, DHP-2, a pharmacological inhibitor of the upstream MAP3K ZAK, blocked Stx2-mediated activation of JNK and p38MAPK, partially protected cells from apoptosis and partially reduced caspase-3 activation without altering protein synthesis inhibition caused by the toxin [93]. Thus, ZAK appears to specifically link signals generated by Stxs with stress-activated protein kinases and apoptosis.

Using human macrophage-like cells in various stages of maturation, the anti-apoptotic factor Bcl-2 was shown to be a crucial mediator of apoptosis or cell survival following Stx intoxication. Enhanced protein and mRNA expression of Bcl-2 were associated with protection from apoptosis induced by Stx1 in toxin-resistant macrophage-like cells under ER stress, while Bcl-2 expression was decreased in toxin-sensitive monocytic cells, leading to rapid apoptosis in the presence of the toxin [111]. Furthermore, amino acid Ser^7^^0^ of Bcl-2 was phosphorylated, and the protein failed to translocate to mitochondria following Stx1 treatment of macrophage-like cells. In contrast, the phosphorylation of Bcl-2 at Ser^7^^0^ was significantly reduced in toxin-treated monocytic cells [111]. The full or potent anti-apoptotic function of Bcl-2 requires JNK-mediated phosphorylation of Bcl-2 at Ser^7^^0^ [112,113]. Thus, Bcl-2, a critical regulator controlling the onset of apoptosis in response to Stx1, was differentially phosphorylated by intoxication. These data suggest that the activation of MAPKs via the ribotoxic stress response may “cross-talk” with the UPR induced by Stxs to regulate Bcl-2 protein expression and activation, which may, in turn, ultimately control apoptosis or cell survival. Although mature macrophage-like THP-1 cells are relatively resistant to the rapid induction of apoptosis by Stxs, downstream signaling through the apoptosis-inducing receptor-ligand pair DR5-TRAIL during ER stress contributes to delayed apoptosis detected in Stx1-treated macrophage-like THP-1 cells [114].

Although apoptotic signaling by Stxs has been observed in many different cell types, there remains much uncertainty about the precise mechanisms by which Stxs activate apoptosis. Ikeda *et al.* [115] found increased intracellular calcium levels and prolonged activation of p38MAPK when Vero cells were treated with Stx1 or Stx2. In this study, treatments with inhibitors of Ca^2+^-mediated signaling (BAPTA-AM, calcium chelator) and inhibitors of p38MAPK partially protected the cells from intoxication. Treatment of the laryngeal epithelial cell line HEp-2 with Stx1 and Stx2 increased the expression of the pro-apoptotic protein Bax without altering the level of pro-apoptotic Bak and anti-apoptotic Bcl-2 expression [116]. Moreover, overexpression of the pro-survival protein Bcl-2 protected the cells from Stx2-induced apoptosis. Using confocal fluorescence microscopy with fluorescein isothiocyanate-conjugated anti-Bax antibody and MitoTracker Red, Lee *et al.* observed that Stx1 was involved in triggering mitochondrial translocation of the pro-apoptotic Bcl-2 family protein Bax [111]. Stx-induced apoptosis in the HeLa cell line (cervical adenocarcinoma cells) was extensively characterized by Fujii *et al.* [117]. They found that apoptosis induction required toxin enzymatic activity and was characterized by activation of caspases-3, -6, -8 and -9. Peptide-mediated inhibition of caspases-3, -6 and -8 blocked apoptosis. In human dermal microvascular and pulmonary arterial endothelial cells, proteasome-mediated degradation of anti-apoptotic Bcl-2 family member Mcl-1 preceded apoptosis (as measured by caspase-3 activation) induced by Stx1 and Stx2, suggesting a crucial role for Mcl-1 in apoptotic signaling in toxin-treated endothelial cells [118]. A different mechanism of signaling in Stx1-induced apoptosis was observed using Burkitt’s lymphoma (BL) cells. BL cells are thought to represent transformed counterparts of normal centroblasts and display high expression of the B-cell differentiation antigen CD77 (Gb_3_). Stimulation of Ramos BL cells with Stx1 resulted in rapid apoptosis with 50% reduction in viability within 9 h [119]. Rapid cleavage of pro-caspase-8, beginning 2–4 h after toxin exposure, was reported to occur coincident with the degradation of c-FLIPL in Stx1-treated BL cells [94]. Apoptosis was not only induced when BL cells were incubated with Stx holotoxin, but was also induced when BL cells were exposed to recombinant toxin B-subunits or immobilized anti-Gb3/CD77 monoclonal antibody, thereby dissociating an apoptotic pathway induced by toxin enzymatic activity from a pathway induced by Gb_3_ cross-linking [120,121].

Less is known about the apoptosis signaling mechanisms activated by Stxs *in vivo*. Apoptotic death was noted in renal tubular cells on biopsy specimens obtained from a child with Stx-mediated HUS [122], and renal biopsies from HUS patients infected with STEC revealed extensive damage to glomeruli with evidence of apoptotic changes and DNA fragmentation [123]. In the mouse model of Stx-mediated renal damage, Stx2 targeted murine collecting duct epithelium with evidence of toxin-induced loss of function contributing to renal failure through the induction of apoptosis in Gb_3_^+^ murine renal cortical and medullary tubular cells [124]. Recently, in order to monitor HUS disease progression utilizing a system more closely resembling the human kidney, DesRochers *et al.* [125] investigated the effect of Stx2 on a bioengineered three-dimensional model of human renal tissue. They observed similar aspects of cytotoxicity following toxin exposure as had been described in two-dimensional cell culture [125]. Numbers of apoptotic monocytes and neutrophils were significantly elevated in the circulation of HUS patients, and leukocyte cell death positively correlated with disease severity as monitored by admission to the intensive care unit [33]. The precise location of Gb_3_ in the mouse and human CNS is controversial, and unlike Stx-mediated renal programmed cell death, apoptotic neuropathogenesis induced by Stxs has not been extensively examined, although rabbit neurons appear to be susceptible to Stx-induced apoptosis [126]. The identification of intermediate signaling molecules in Stx-induced apoptosis may represent therapeutic targets to intervene in toxin-induced cell death, thereby ameliorating tissue damage caused by Stxs [127]. However, many details on apoptotic signaling pathways initiated by Stxs remain to be clarified.

### 3.4. Autophagy

Autophagy is a catabolic process originally defined as providing survival signals for cells undergoing nutrient deprivation. During autophagy, a double membrane forms around damaged organelles or intracellular substrates to generate an autophagosome. This double-membraned vesicle may then fuse with late endosomes or lysosomes to mediate the degradation of the inner membrane and autophagosomal contents. However, it is now understood that depending on the cell type and the type of stress encountered, autophagy may antagonize or facilitate apoptosis or necrosis [128,129,130,131]. For example, while nutrient deprivation leads to autophagy, the removal of damaged organelles and cell survival, if cellular homeostasis is not restored, then delayed apoptosis may follow. Booth *et al.* [132] suggested that there is regulation of apoptosis by autophagic protein partners and *vice versa* by “cross-talk” between the two pathways. Based on the observation that treatment of MDCK or Vero cells with autophagy inhibitors, such as 3-MA, protected cells from apoptosis induced by the ribosomal-inactivating proteins Stx and ricin, autophagy signaling may be necessary for Stxs to induce cell lysis in toxin-sensitive cells [133]. While more work remains to be done to fully elucidate the direct effects of Stxs on autophagy, it is now known that Stx-induced signaling pathways leading to the induction of autophagy may activate apoptosis or cell survival programs conferring the toxin-sensitive or toxin-resistant phenotypes, respectively [134] (Figure 2). Lee *et al.* [134] observed that in toxin-resistant primary human macrophages, Stxs are translocated to lysosomes, and autophagy is induced in the absence of calpain and caspase activation, as well as Atg5 and Beclin-1 cleavage. In contrast, using toxin-sensitive cells, Stxs are translocated to the ER; the ER stress response is activated; and autophagy is induced in association with the activation of calpains and caspase-8 and-3, as well as the cleavage of Atg5 and Beclin-1. Recent studies support a role for autophagic signaling in Stx-mediated cytotoxicity. Tang *et al.* [106] showed that treatment of intestinal epithelial cells with Stx2 initiated autophagic cell death via the ER stress pathway and by triggering pseudokinase TRIB3-mediated DDIT3 expression and AKT1 dephosphorylation. It is interesting to note that in contrast to studies supporting a role for autophagy in cell death, autophagy may attenuate ER stress through the sequestration and degradation of unfolded proteins [130,135,136]. Given that autophagy may coincide with apoptosis and promote cell death, or antagonize apoptosis to promote cell survival, further studies are warranted to elucidate precise mechanisms by which autophagy induced by Stxs facilitates or inhibits apoptosis.

### 3.5. Inflammatory Response

The depurination of the ribosome by the enzymatic A_1_-fragment of Stxs is a crucial event in the activation of host signal transduction pathways leading to the proinflammatory response [87,137,138,139]. Inflammatory responses include, in part, the synthesis and secretion of cytokines and chemokines by immune cells following the detection of “danger signals”, such as bacterial cell membrane or cell wall components, toxins or flagellins [140,141]. The regulated elicitation of inflammation leads to the elimination of microbes or foreign agents. However, excess inflammation may lead to harmful effects, including septic shock, organ failure or tissue damage [142,143]. Multiple studies have suggested that the innate immune response is activated by Stxs, which, in turn, play a role in disease progression and/or tissue injury by inducing the increased expression of genes involved in the biosynthesis of the toxin-binding glycolipid Gb_3_. Thus, the production of pro-inflammatory cytokines in response to the toxins may sensitize microvascular endothelial cells found in target organs to the cytotoxic action of Stxs [144,145,146]. In 1995, Raqib *et al.* [147] measured proinflammatory cytokine levels in plasma and stool obtained from patients infected with *S. dysenteriae* serotype 1 or non-toxigenic *S. flexneri*. Levels of cytokines detected in stool were approximately 100-times higher in stool than plasma at the onset of diarrhea. Furthermore, they observed that levels of the pro-inflammatory mediators TNF-α, IL-8, IL-1β, IL-6 and GM-CSF directed into the stool were significantly higher in patients infected with Stx-producing *S. dysenteriae* serotype 1 than in patients infected with *S. flexneri*. In sera from HUS patients infected with *E. coli* O157:H7, levels of IL-6, IL-8, IL-10 and endothelin were significantly elevated compared to those patients infected with *E. coli* O157:H7 experiencing colitis only [148]. The cellular sources of the elevated cytokines detected in the circulation of HUS patients are not known. Several studies support the concept that macrophages may be the source of cytokine production to promote tissue damage through increased toxin receptor expression and/or leukocyte recruitment. When monocytic THP-1 cells were differentiated into the macrophage-like state, the cells became relatively resistant to the cytotoxic action of Stxs, but also became capable of secretion of TNF-α, IL-1β, IL-8, MIP-1α, MIP-1β, MCP1 and Groβ [138,139,140]. Stx-induced cytokine expression was manifested in the presence or absence of lipopolysaccharide (LPS) [87,139,149,150,151]. Furthermore, non-adherent primary human monocytes cultured in Teflon foil bags were found to respond to Stx1 or Stx2 by producing TNF-α and GM-CSF without undergoing apoptosis, suggesting that peripheral blood monocytes may be a cellular source of the cytokines in the circulation [152]. Several studies have reported that despite expressing toxin receptor Gb_3_, human monocytes/macrophages are relatively insensitive to the cytotoxic action of Stxs compared to other Gb_3_^+^ cell types, which are highly toxin-sensitive [134,153,154]. Monocyte insensitivity to Stxs appears to correlate with the failure of Gb_3_ to associate within lipid rafts, which may be necessary for efficient retrograde transport of Stxs [155]. Taken together, these data suggest that infiltrating macrophages may produce tissue factors and cytokines in response to Stxs that further augment inflammation, thrombogenesis and tissue damage to ultimately lead to organ failure [156]. Multiprotein complex inflammasome function in Stxs-induced inflammatory cytokine expression and its role in host cell death have been incompletely understood. Very recently, Lee *et al*. have reported that Stxs induce NLRP3 inflammasome activity, resulting in cleaved caspase-1 to release active proinflammatory cytokine IL-1β and promoting apoptotic cell death by increasing caspase-3 activity [157].

Evidence for energy-requiring transcytotic and/or paracellular Stx1 and Stx2 epithelial transport systems has been presented [158]. The innate immune response may facilitate the chemotactic infiltration of inflammatory cells into the lamina propria of the gut and, thus, mediate cell morphology changes in the intestinal epithelial barrier, which allow Stxs to cross into lamina propria to damage colonic blood vessels to initiate hematogenous spread [28]. In intestinal cell lines T84 and HCT-8, it has been demonstrated that Stxs play a role as mediators of intestinal inflammation for the induction of IL-8 protein and cytokines in response to the toxin may facilitate intestinal colonization [159,160]. It has become increasingly apparent that Stxs as multifunctional signaling molecules activate cell stress responses and may contribute to the toxin-induced inflammation, in addition to the capacity of the toxin to mediate adenine depurination from the rRNA backbone. Stx activation of MAP3K ZAK through the ribotoxic stress response results in activation of MAP2Ks that subsequently phosphorylate MAPK JNK and p38 along with activation of ERK1/2 via phosphorylation of MEK1/2 in promoting intestinal epithelial inflammation by inducing IL-8 mRNA expression [160]. Of note, the initiation of intestinal inflammation in hemorrhagic colitis during the infection with Shiga toxin-producing bacteria is multifactorial, with other microbial products participating in the induction. For example, Miyamoto *et al.* treated human intestinal epithelial Caco-2 cells with purified H7 flagellin in the presence or absence of Stx2 and showed that flagellin signaling through TLR5 may be a relatively more potent inducer of IL-8 production in an *in vitro* model system [161].

Following the entry of Stxs across the intestinal epithelial barrier into the bloodstream, a primary target organ for vascular damage and failure is the kidney. Using a murine model of Stx-mediated renal damage, macrophages were recruited to the kidneys of mice challenged with Stx2, and elevated levels of chemoattractants, such as MIP-1α, were detected in the kidneys [162]. Using a non-human primate model of HUS, after intravenous injections with Stxs, the baboons developed progressive thrombocytopenia, HUS with signs of glomerular thrombotic microangiopathy and systemic inflammatory responses in the baboon kidney, small intestine, colon, lung and spleen, with striking chemotactic profiling of highly expressed mRNAs for IL-8, MCP-1 and MIP-1α [163,164]. The regulation of cytokine and chemokine expression by Stx-treated macrophages appears to be quite complex. In addition to activating MAPK cascades, Cherla *et al.* [165] demonstrated that signaling through the PI3K/Akt/mTOR pathway initially activated cytokine expression via the phosphorylation of the eukaryotic translation initiation factor 4E-BP, but then negatively regulated proinflammatory cytokine production through the phosphorylation (inactivation) of the positive regulator of cytokine production, glycogen synthetase kinase (GSK)-3. These data suggest that embedded within the Stx-induced signaling mechanisms enhancing cytokine production are signals that will ultimately down-regulate cytokine production and return the host to a normal homeostatic state. In addition to the tight regulation of the proinflammatory response, the timing of exposure to Stx1 or Stx2 *vs.* cytokine production has been shown to be critical in the outcome of disease *in vivo*. Mice receiving TNF-α for 1 h prior to toxin exposure were protected from lethality, while mice receiving TNF-α after the administration of toxins displayed increased lethality associated with increased glomerular damage [166]. Using immortalized adult human proximal renal tubular epithelial cells, HK2, treatment with Stx2 selectively produced two chemokines MIP-1α (CCL3) and MIP-1β (CCL4) that were not induced by Stx1 treatment [104]. Although Stxs are primary pathogenic virulence factors for HUS development, in severe cases of HUS, the toxin-mediated damage is not limited to the kidney, but may extend to the brain as another important target for the toxin after entering systemic circulation. In human brain microvascular endothelial cells (HBMEC) and human brain endothelial cells (HBEC), pretreatment with TNF-α and IL-1β as inflammatory stimuli markedly increased the toxicity of Stxs by enhancing the expression of the toxin receptor Gb_3_ on these cells in comparison with the untreated cells, suggesting the role of proinflammatory cytokines in sensitizing brain cells to the toxins [145,146,167]. In the 2011 outbreaks of Shiga toxin-producing EHEC infections in Germany, these findings were confirmed in the brain of infected patients with HUS and neurological complications as a consequence of systemic inflammatory cascades activated by upregulating CD77/Gb_3_ neuronal expression [168]. Importantly, a rabbit model treated with purified Stx2 showed that neuroinflammatory responses, such as increased expression of TNF-α or IL-1β in the CNS parenchyma and microglial activation at an early stage, may lead to occasional apoptotic neurons as the onset of neurological symptoms later in the disease [126]. The unanswered question regarding the precise mechanism of Stx-mediated neurological damage highlights the importance of inflammatory mediators or the permeability of brain endothelial cells to influence blood-brain barrier (BBB) function once Stxs reach brain parenchyma. Landoni *et al.* demonstrated that Stx1-induced secretion of TNF-α on LPS-stimulated astrocytes, inflammatory cells in the brain, affected the integrity of brain endothelial cells and maintenance of BBB properties to contribute to the development of neuropathological symptoms observed in HUS [169].

Histologic examination of animals revealed that monkeys receiving the toxin-producing strains had damage to colonic capillaries within the lamina propria and inflammatory vasculitis of peritoneal mesothelium. Thus, the production of Stx was associated with increased vascular endothelium damage. We and others showed that human endothelial cells derived from a number of anatomical sites became more sensitive toward Stx cytotoxicity when they were simultaneously treated with the toxins and proinflammatory cytokines TNF-α or IL-1β [145,170,171]. Van de Kar *et al.* showed that pre-exposure of endothelial cells to both TNF-α and IL-1β upregulated the expression of membrane Gb_3_ on the cells *in vitro* [144]. However, endothelial cells *in vitro* appeared to be relatively refractory to the cytotoxic action of Stxs [145]. We also showed that the administration of a single dose of murine TNF-α after Stxs administration in mice dramatically affected renal pathology, so that glomeruli showed increased vascular damage [166]. Collectively, these data suggest that the innate immune response elicited by Stxs contributes to the development of vascular lesions by sensitizing endothelial cells to the action of the toxins. Further understanding of how Stxs regulate the expression of pro- and anti-inflammatory effectors, as well as the cytokine and chemokine profiles expressed by specific cell types will be necessary to develop therapeutic agents to ameliorate tissue damage. More information on the signaling mechanisms activated by Stxs to modulate the inflammatory response can be found in the review of Lee *et al.* [172].

## 4. Toxin Engineering for Therapeutics

The use of engineered toxins as cancer therapeutic agents represents one of the most novel approaches in targeted cancer therapy. Stxs are promising candidates in this approach because the toxins have several intracellular processing steps necessary to express toxicity. Thus, host cell targeting factors may be used to activate the engineered Stx molecule (inactivate toxicity or activate the anti-cancer agent). For example, the introduction of a tumor protease binding site in the A-subunit in place of the furin cleavage site would biologically “switch-off” Stx toxicity without affecting B-subunit binding and retrograde transport, delivering the engineered molecule to its intracellular target. Many tumor cells overexpress Gb_3_ on their surface, including tumors of the colon [52,173,174] and breast [175]. It was recently reported that Gb_3_ expression was detected in 17 out of 25 tumors from breast cancer patients [176] and from glioma cells [177]. Intratumoral injection of Stx1 inhibited tumor growth in mouse xenograft models, clearly demonstrating an antineoplastic effect [178]. Stx1 has been applied to several types of tumors, such as fibrosarcoma [53], meningiomas [179] and bladder carcinoma [178]. Xenografts of human renal carcinoma completely regressed with a single intratumoral injection of Stx1 [180], and apoptosis was detected without other side effects in this model [181]. These findings strongly suggest that Stxs could be exploited to target human cancers. However, there are side-effects to overcome using Stxs as targeted cancer therapeutic agents, such as microvascular damage and, in the worst case, the development of HUS or CNS abnormalities.

**Figure 3:**
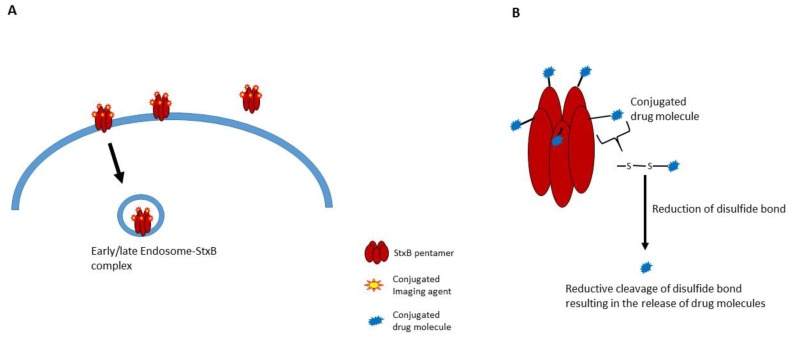
Schematic diagram of StxB-anticancer agent conjugates, imaging molecules (**A**) and chemical drug molecules (**B**) [184].

Non-toxic B-subunits of Stxs may be developed to target cancer cells. Stx B-subunits would, in principal, deliver therapeutic agents into cancer cells. There are several on-going trials to engineer Stxs for cancer targeting and drug delivery, especially in the area of cancer cell detection by using the Gb_3_ receptor and B-subunits labeled with positron emitters [51]. The B-subunits of Stxs have many of the desired molecular characteristics for the delivery of cargo molecules to intracellular sites. These characteristics include stability across a broad pH range, resistance to proteases and the capability of crossing tissue barriers [81]. Janssen *et al.* reported that Stx B subunits conjugated to contrast agents targeted Gb_3_-expressing adenocarcinomas in a transgenic mouse model using different imaging modalities [51] (see the excellent review summarizing Gb_3_ expression levels in primary human cancers [82]). Stx B-subunit and photosensitizer (TPPp-O-β-GluOH)_3_ conjugates killed cancer cells [182]. A seminal study utilizing this approach involved the creation of a B subunit-camptothecin 11 (topoisomerase I inhibitor) conjugate linked by a disulfide bond. This agent was highly cytotoxic for HT29 colorectal carcinoma cells *in vitro* [183]. The most interesting concept explored in this study was to design the conjugate so that the anti-cancer drug was released in the ER after reduction of the disulfide linkage connecting the Stx B subunits with camptothecin 11 [183]. This approach represents an advanced drug payload technique, which may be used in conjunction with antibody-drug conjugates (ADC). Batisse *et al.* reported the successful construction of Stx B subunit-monomethyl auristatin (MMA)/monomethyl auristatin E (MMAE) conjugates that released MMA/MMAE upon reaching the reducing environment in the ER [184] (Figure 3). These conjugates have the potential to eliminate tumor cells overexpressing Gb_3_ on their surface [184]. Likewise, StxB conjugation with contrast agents has the potential to be used for targeted tumor imaging. Fluorophore-labeled StxB was accumulated and detected in digestive cancers *in vivo* after 2.5 h of oral administration. Further, StxB-[^18^F] fluoropyridine-StxB conjugates that were systemically injected significantly improve the efficiency of PET imaging toward digestive tumors in mouse [51]. Viel *et al*. reported that fluorescent StxB conjugates were accumulated around the tumor area and enter the Gb_3_-expressing cancer cells in xenografted nude mice. In addition, they demonstrated a potential of these conjugates for the analyses of tumor at the cellular level with confocal microscopy [185]. Further, the targeted imaging of cancer cells by StxB-functionalized fluorescent microbubbles (micro-sized gas bubbles as ultrasound contrast agents [186]) has been demonstrated. These StxB microbubbles significantly enhanced the intensity of ultrasound imaging *in vitro* and in a tumor xenograft mice model [187]. Based on these findings, StxB-based targeted imaging agents might be a novel diagnosis method for cancer patients in early stages.

In developing personalized cancer therapies, the stability of the therapeutic agents and their targeting specificity still remain as challenges. Despite recent advances in drug delivery technology, non-specific delivery and low efficacy of the agents must be avoided. To enhance specificity, the agents need to be engineered with multi-targeting factors, because non-cancerous cells may also have “tumor targeting receptors” on their surface. In order to achieve this goal by protein engineering, toxins requiring multiple steps for intoxication of target cells have an advantage. Naturally-occurring toxins may require many host cell-specific targeting factors to reach intracellular sites of action. One can engineer these toxins to carry cancer-specific molecules, such as tumor proteases or tumor-specific receptors. Stxs are excellent candidates for anti-cancer drug delivery, because they have at least three well-defined processing steps involved in intoxication that may be manipulated: Gb_3_ binding, proteolysis by furin and reduction of a disulfide bond. Future studies exploring the utility of Stxs as engineered molecules for drug delivery may include alteration of the Gb_3_ binding sites defined on B-subunits to specifically bind receptors uniquely displayed on cancer cells. The alteration of the A-subunit furin binding domain to a tumor protease binding domain would enhance targeting specificity. Reduction of the disulfide bond could potentially release the anti-cancer agent once inside the target cells.

## 5. Conclusions

Infections with Shiga toxin-producing bacteria continue to be a significant world-wide public health concern. Advances in clinical treatment have primarily been limited to supportive therapies, and prevention has focused on improved education of the public on the risks of ingesting contaminated food or water. Shiga-toxin producing bacteria are still a significant cause of morbidity and mortality, and the development of vaccines and effective interventional therapies is clearly needed. Shiga toxins comprise a family of genetically- and functionally-conserved cytotoxins produced by the enteric pathogens *Shigella dysenteriae* serotype 1 and an increasingly expanding group of *Escherichia coli* serotypes [188]*.* The purification and subsequent elucidation of the crystal structures of the Stxs were critical in formulating structure/function studies defining toxin binding, intracellular routing and processing and the mode of action [188]. It has become increasingly apparent that while Stxs are potent protein synthesis inhibitors, they are also multifunctional proteins capable of activating signaling pathways initiated by the ribotoxic stress response and ER stress, leading to apoptosis, autophagy and/or cytokine/chemokine production. Stx-induced signaling pathways may contribute to damage in the colon and the development of life-threatening conditions, such as acute renal failure (HUS) or neurological disorders. Studies to identify key signaling molecules most necessary for promoting damage to the host are needed. Since the ribotoxic stress response and ER stress elicited by Stxs have the ability to activate apoptosis, autophagy or proinflammatory cytokine/chemokine production, blocking the proximal sensors of stress or their downstream effector molecules potentially represent therapeutic approaches to treat illnesses caused by Stxs. However, Stxs may induce different signaling pathways in different cell types, which may contribute to cell death or cell survival. An improved understanding of intermediate signaling molecules involved in influencing both Stx-mediated toxicity and pathogenic effects may provide researchers with potential targets to intervene in toxin-induced disease progression. Further, engineering Stxs may be a promising approach to develop novel anticancer therapeutics with high targeting specificity and therapeutic efficacy.
